# Assessing the effect of regular swimming exercise on the micro- and macrovascular physiology of older adults (ACELA II study)

**DOI:** 10.3389/fphys.2023.1223558

**Published:** 2023-09-12

**Authors:** M. Klonizakis, A. Mitropoulos

**Affiliations:** Lifestyle, Exercise and Nutrition Improvement (LENI) Research Group, Sheffield Hallam University, Sheffield, United Kingdom

**Keywords:** flow-mediated dilation, endothelial function, cardiovascular disease, aquatic exercise, water-based exercise

## Abstract

**Introduction:** Cardiovascular disease (CVD) remains the main cause of death in the Western world. Our recent findings demonstrate potential CVD risk reduction in older adults who undertake regular swimming exercise. Nevertheless, it remains unknown whether an exercise intervention based on swimming is feasible and effective prior to a wider implementation of a CVD risk prevention strategy.

**Methods:** This was a pragmatic, two-group, randomised controlled trial. A total of 40 older adults were randomly split into two groups (*n* = 20 per group). The swimming exercise group consisted of participants who engaged in swimming exercise (2–3 days/week, for 8 weeks). The control group did not perform any exercise. Flow-mediated dilation (%FMD) was the primary outcome. Secondary outcomes included raw cutaneous vascular conductance. Feasibility outcomes (e.g., recruitment, adherence, and attrition rates) were also assessed.

**Results:** Statistically significant macrovascular (%FMD; swimming group: 9.8% ± 4.2%, *p* <0.001; control group: 4.6% ± 2.5%) and microvascular function (raw cutaneous vascular conductance; swimming group: 4.1 ± 0.9, *p* <0.01; control group: 3.2 ± 1.1) improvements were observed in the swimming group compared to the control group. Compliance to twice and thrice weekly in an 8-week swimming exercise was 92.6% and 88.4%, respectively, with no dropouts.

**Conclusion:** Our 8-week, community-based, pragmatic swimming exercise intervention is a feasible and effective exercise programme that could be implemented in older adults for the prevention of age-related CVD. These findings suggest that swimming exercise could significantly reduce CVD risk in older adults, and a large research clinical trial is warranted to establish these findings.

## Background

Cardiovascular disease (CVD) remains the main cause of death in the Western world (Townsend et al., 2022), severely affecting the quality of life (QoL) of those who suffer. Of particular interest among high-CVD-risk groups are older people (>55 years of age), as cardiovascular (CV) ageing affects pathophysiological pathways, which are also implicated in CVD development.

Exercise may delay CV ageing and consequently prevent the development of CVD in older people (Lee et al., 2012). However, unsurprisingly, land-based exercise participation and adherence in older populations are low and decrease further with age (Public Health England Team, 2022). There are several barriers for older people to exercise, with concerns of injury and lack of confidence in using exercise equipment often being cited as significant barriers to participation (Spiteri et al., 2019). Therefore, it is important to develop exercise interventions based on forms of exercise that older people feel comfortable with; water-based exercise may meet this need, as it remains popular among older people, albeit with a downward trend (Swim England, 2022).

Our group recently completed a cross-sectional observational study (Klonizakis et al., 2020) to compare the micro- and macrocirculatory function in groups of “older-but-healthy” adults, undertaking different modes of long-term, aerobic exercise regimes. The comparison included adults undertaking regular aquatic exercise and others following regular, low-intensity interval training, land-based exercise, as well as adults following a mixture of both land- and water-based exercise. Our findings demonstrated a beneficial CVD risk reduction in older adults who undertake regular land-based exercise (Lanting et al., 2017), as well as those who follow a regular aquatic-based exercise programme, irrespective of whether this is conducted on its own or in combination with land-based exercises.

Nevertheless, the current literature presents limited evidence on the wider CV benefits for those who engage in aquatic exercise. Some studies show no improvement in CV parameters (Porter et al., 2019), while others suggest a positive effect on the intima-media thickness of carotid arteries, haemodynamic and biochemical markers (Farinha et al., 2022), and aortic haemodynamics (Fukuie et al., 2021). Therefore, a research study exploring both CVD risk reduction indices and feasibility parameters (e.g., assessing whether wider implementation of a water-based programme is possible) in a controlled RCT study environment is warranted.

To address this knowledge gap, we conducted ACELA II, in which we carried both micro- and macrovascular assessments in people randomised to follow a swimming exercise intervention for an 8-week period, as well as collecting information on feasibility parameters. Our findings are presented as follows.

## Methods

### Study design

This was a single-centre, pragmatic, two-arm randomised controlled trial conducted in Sheffield, United Kingdom, in succession of the ACELA study (Klonizakis et al., 2020); recruitment took place between November 2019 and February 2022. Recruitment was paused during periods when access to swimming pools in England was not allowed due to the COVID-19 pandemic (March 2020–August 2020 and November 2020–May 2021). Participants were recruited via social and mass media (Twitter, Facebook, and newspaper advertisement), posters (in community venues and halls, Hallam University, places of worship, and post offices), open e-mail invitation, and word of mouth.

General inclusion criteria included being over 55 years of age and normotensive (e.g., <140/90 mm Hg) and have at least basic swimming ability. Participants should also have been undertaking less than 60 min of structured/planned physical activity per week for at least 6 months. Exclusion criteria included having any overt chronic disease (e.g., severe lung or heart disease) which would affect vascular functionality (i.e., diabetes mellitus and coronary heart disease), anaemia (irrespective of whether an iron supplementation course was followed or not), and recent (e.g., in the past 3 months) major surgery. Participants undertaking high-intensity interval training of any form, at the time of recruitment, were excluded.

The study was approved by the Ethics Committee of the Faculty of Health and Wellbeing of Sheffield Hallam University (ER5320861). All participants provided informed consent prior to their participation in the study.

## Study procedures

Following the provision of informed consent and habituation with the study procedures (supported by a member of the research team not involved in data collection), the participants were invited to Sheffield Hallam University for two visits.

### Anthropometrics and quality of life

During visit 1, anthropometry measures (e.g., stature, body mass, and waist and hip circumferences), demographic data (e.g., age and gender), and smoking and clinical history (e.g., previous operations, comorbidities, and current medications) were collected. The participants were requested to complete the QRISK (Collins and Altman, 2010), International Physical Activity Questionnaire (IPAQ), and EQ5D-5L (Herdman et al., 2011) (including a health-related visual analogue scale (VAS)) questionnaires to assess the risk of cardiovascular disease, physical activity levels, and quality of life, respectively.

### Macrovascular function

Our primary outcome was macrovascular function, assessed using flow-mediated dilation (FMD), and is presented as change in the post-stimulus diameter as a percentage of the baseline diameter (%FMD). Reduced altered brachial artery FMD is an early marker for endothelial dysfunction, which is considered a predictor for long-term, adverse cardiovascular events (Messner and Bernhard, 2014). FMD is a noninvasive, nitric oxide-mediated measure. Baseline scanning to assess the resting vessel diameter was recorded over 3 min, following a 10-min resting period, using a SONIMAGE MX1 ultrasound machine (Konica Minolta, Tokyo, Japan), according to the International Brachial Artery Reactivity Task Force guidelines (Corretti et al., 2002). Fixed anatomic landmarks (side branches) and a stereotactic adjustable probe-holding device were used, ensuring that the same scan was maintained throughout the study. The technical error in our laboratory for FMD is 5% (Klonizakis et al., 2022).

### Microvascular function

Microcirculatory function (Wasilewski et al., 2016) was also assessed. Skin microvasculature is an established assessment of microvascular function (Wasilewski et al., 2016). The participants were instructed to abstain from caffeine consumption 2–3 h prior to the recordings to eliminate the confounding effect caffeine may have produced on vasorelaxation. Skin blood flow was measured as cutaneous red blood cell flux using a laser Doppler flowmeter. Local thermal hyperaemia was induced using a heating disc surrounding the probe. The probe was attached to the skin using a double-sided adhesion sticker and was placed at the anterior view of the forearm between the elbow and the wrist joints. The laser Doppler signal was recorded using PeriSoft Windows 9.0 software (PSW 9.0). The participants were rested in a supine position in a temperature-controlled room with a constant ambient temperature of 24° for 35 min. The heart rate and diastolic and systolic blood pressure were recorded from the left arm at 5-min intervals throughout the protocol (DINAMAP Dash 2500, GE Healthcare, United States). Baseline skin blood flow data were recorded for 5 min with the local heating disc temperature set at 30°. Following this, rapid local heating was initiated to obtain maximal vasodilation, and the temperature was increased by 1° every 10 s until 42° was reached. This was then maintained for 30 min, following which the test was completed. Measurements of red cell flux (recorded in arbitrary units (AU)) were divided by the corresponding mean arterial pressure (MAP) values (in mmHg) to obtain CVC in AU/mmHg. The different phases during the LDF assessment are described in Table 1.

**TABLE 1:** Laser Doppler flowmetry measurement phase definition.

Measurement phase	Time point
Baseline	Arithmetical mean of the last 2 min of the first 5-min period
Initial peak	Arithmetical mean of the highest consecutive 30-s period within the district initial hyperaemic response
Plateau	Arithmetical mean of the last 2 min of heating at 42°C
Maximum	Arithmetical mean of the last 2 min of heating at 44°C

### Randomisation

Following baseline assessments, the participants were randomised remotely (to ensure allocation concealment) into group A (undertaking water-based exercise) or group B (no intervention). The randomisation was performed by an independent statistician using a computer programme (nQuery Advisor 6.0, Statistical Solutions, Ireland) to generate block-randomisation. Each participant was allocated a unique trial number.

### Intervention

Group A participants received a 12-week, complimentary pass allowing them to undertake swimming in one of our six collaborating leisure centres at Sheffield (i.e., Concord, Ponds Forge, Hillsborough, Heeley, Springs, and Westfield). These were chosen to ensure the widest possible accessibility for our participants and to test the feasibility of a pragmatic, self-managed, community-based exercise intervention. Their basic swimming ability was confirmed, and the participants were instructed to undertake between two and three swimming sessions per week for approximately 45 min each session. The participants were instructed to perform a self-pace swimming session based on individual capabilities, including intensity and resting periods. The participants were free to perform any stroke throughout the swimming session. Regularity of attendance was monitored and self-reported by participants. Our original plan to include water-based exercise classes was not possible due to COVID-19 restrictions.

To support our study participants, we also used our “six pillars of adherence” framework (based on “social support,” “education,” “reachability,” “small group intervention implementation,” “reminders,” and “simplicity”), which has been proven successful in previous studies (Wasilewski et al., 2016; Mitropoulos et al., 2020; Klonizakis et al., 2022). These included bimonthly support phone calls and regular e-mails to stimulate attendance.

To maintain equality, group B participants were also offered a complimentary pass to the same leisure centres, following their follow-up assessments.

### Follow-up assessments

The participants were invited for their follow-up assessments 8 weeks after their first exercise session (group A) or after their baseline assessments (group B). All baseline assessments were repeated at that stage.

### Bias and study size

Evaluators were blinded to participant grouping to reduce investigator bias. Additionally, to decrease inter-observer bias and increase measurement consistency, each evaluator carried out the same measurement for each participant. That is, one undertook the microcirculatory assessment each time, while another undertook the macrocirculatory assessment each time.

## Statistical analysis

Normality of each of the outcome variables was assessed using the Shapiro–Wilk test. All of our outcomes were parametric. Data were analysed in SPSS 24 (IBM U.K. Limited, Hampshire, United Kingdom) on an intention-to-treat basis using group average data to impute missing data at 8 weeks. Differences between the two groups were determined by independent t-tests and within groups (i.e., pre- and post-assessment) with paired sample t-tests. Statistical significance for the test was set at *p* ≤ 0.05. Values are presented as the mean ± standard deviation (SD), unless otherwise stated.

Feasibility data were also collected to support future implementation of the intervention. Recruitment rates were measured as the rate of invited participants who were eligible and consenting. Acceptability of allocation was assessed by examining reasons for dropout in discontinuing participants and comparing attrition rates between the two study groups. Suitability of measurement procedures was evaluated by outcome completion rates and reasons for missing data. The attrition rate was established as the discontinuation of intervention and loss to follow-up measurement for all conditions. The acceptability of the exercise programmes was assessed by using session attendance and compliance data. The safety of exercise training was also assessed by exploring reasons for dropout from the exercise programme and the number and type of adverse events that occurred in each group.

## Results

### Participants

Participant characteristics did not present any differences between the groups both at baseline (Table 2) and follow-up assessments. However, within-group differences demonstrated that the waist circumference improved in the exercise group (88.3 ± 15.6 cm, *p* <0.001) after exercise intervention, whereas the waist (93.2 ± 11.5 cm, *p* <0.05) and hip circumferences (104.8 ± 8.8 cm, *p* <0.01), as well as the percentage of body fat (37.0% ± 6.4%), were increased for the control group after the 8-week period.

**TABLE 2:** Subject characteristics at baseline assessments.

	Baseline		Follow-up	
	Aqua (*n* = 20)	Control (*n* = 17)	Aqua (*n* = 20)	Control (*n* = 17)
Sex	12 F/8 M	10 F/7 M		
Age (years)	61 ± 4	62 ± 5		
Body mass (kg)	83 ± 15	75 ± 14	80 ± 11	76 ± 13
Body fat (%)	36.7 ± 9.0	36.2 ± 6.5	34.4 ± 10.5	37 ± 6.4
BMI (kg m^-2^)	27.7 ± 8.6	27.2 ± 4.2	29.7 ± 10.2	27.5 ± 4.1
Waist circumference (cm)	93.8 ± 15.4	91.5 ± 11.6	88.3 ± 15.6***	93.2 ± 11.5*
Hip circumference (cm)	108.6 ± 10.6	103.8 ± 9.4	101 ± 13.4	104.8 ± 8.8**
WHR	0.89 ± 0.09	0.88 ± 0.07	0.85 ± 0.09*	0.89 ± 0.08
Systolic BP (mmHg)	129 ± 20	128 ± 17	127 ± 19	128 ± 20
Diastolic BP (mmHg)	78 ± 10	77 ± 12	76 ± 12	78 ± 15
MAP (mmHg)	98 ± 12	100 ± 9	98 ± 10	100 ± 13
IPAQ (METS/week)	1,100 ± 400	1,151 ± 357	1,564 ± 352**	1,120 ± 402
Q-risk (%)	15 ± 3	14 ± 6	14 ± 2	14 ± 4

*p <0.05; **p <0.01; and ***p <0.001. Data are presented as the means ± SD. BMI: body mass index; BP: blood pressure; IPAQ: International Physical Activity Questionnaire; MAP: mean arterial pressure; WHR: waist-to-hip ratio.

## Macrovascular assessment

**FIGURE 1:**
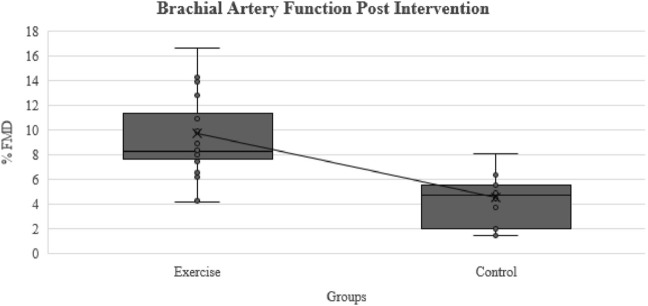
Comparison of brachial artery function (FMD%) after exercise intervention between the exercise and control groups.

Macrovascular function, which was assessed via the FMD coupled with an ultrasound, did not show any differences between the exercise (4.9% ± 2.9%) and control (4.8% ± 2.2%) groups at baseline. Nevertheless, following the exercise intervention, the macrovascular function was significantly improved in the exercise group (9.8% ± 4.2%, *p* <0.001) compared to the control group (4.6% ± 2.5%), as shown in Figure 1. The exercise group presented within-group differences in the pre-intervention arterial diameter post-occlusion (3.93 ± 0.6) compared to the post-intervention arterial diameter post-occlusion (4.2 ± 0.5, *p* <0.01). Similarly, in the exercise group, the %FMD improved from baseline (4.9 ± 2.9) to post-intervention (9.7 ± 4.3, *p* <0.001). There were no sex differences in the %FMD responses both at baseline and post-intervention.

## Microvascular assessment

Statistically significant differences were found in the between-group comparison for the LDF follow-up assessment, as presented in Table 3. The exercise group also presented within-group improvements post-intervention for the initial peak (2.3 ± 0.8 vs. 2.6 ± 0.8, *p* <0.05), plateau CVC (87.3 ± 34.5 vs. 85.3 ± 10.7, *p* <0.05), and CVC max (3.2 ± 1.0 vs. 4.1 ± 0.9; *p* <0.001). The control group presented a decrease in the CVC baseline (0.35 ± 0.1 vs. 0.25 ± .01; *p* <0.05). There were no sex differences for all LDF parameters both at baseline and post-intervention.

**TABLE 3:** Between- and within-group comparison of micro- and macrovascular function and quality of life outcomes pre- and post-intervention.

	Group			
	Baseline		Follow-up	
	Aqua	Control	Aqua	Control
CVC max (%)				
Baseline	8.73 ± 6.5	12.6 ± 9.1	10.7 ± 4.2*	7.9 ± 3.3
Initial peak	68.3 ± 8.7	74.1 ± 17.6	74.4 ± 18.1	66.6 ± 12.6
Plateau	87.3 ± 34.5	84.2 ± 21.0	85.3 ± 10.7*	81.2 ± 10.1
RAW CVC (APU/mm Hg)				
Baseline	0.30 ± 0.2	0.35 ± 0.1	0.29 ± 0.1	0.25 ± 0.1*
Initial peak	2.3 ± 0.8	2.3 ± 0.7	2.6 ± 0.8*	2.2 ± 0.8
Plateau	2.7 ± 0.5	2.7 ± 1.1	3.06 ± 0.9	2.6 ± 1.1
Max	3.2 ± 1.0	3.1 ± 0.78	4.1 ± 0.9***	3.2 ± 1.1
EQ-5D-5L				
Mobility	1.2 ± 0.4	1.1 ± 0.2	1.14 ± 0.4	1.06 ± 0.2
Self-care	1.05 ± 0.2	1.0 ± 0.0	1.0 ± 0.0	1.0 ± 0.0
Usual activity	1.25 ± 0.6	1.24 ± 0.4	1.07 ± 0.3	1.24 ± 0.4
Pain	1.65 ± 0.7	1.59 ± 0.5	1.57 ± 0.6	1.59 ± 0.4
Anxiety	1.1 ± 0.3*	1.41 ± 0.5	1.07 ± 0.3***	3.0 ± 0.7***
VAS	75.6 ± 10.1	76.1 ± 4.5	81.2 ± 11.5*	72.8 ± 3.1

*p <0.05; **p <0.01; and ***p <0.001. Values are expressed as the mean ± standard deviation; CVC: cutaneous vascular conductance; APU: arbitrary perfusion units; CVC max (%): percentage of maximum CVC; aqua: aquatic exercise; VAS: visual analogue scale.

## Feasibility outcomes

### Recruitment rates

Of the 148 people who were approached for participation, 92 met the eligibility criteria and were invited. From those invited, 40 were recruited (21 women and 19 men), and the remaining 52 declined participation for various reasons, such as COVID-19 social restrictions and personal concerns (*n* = 23), travel to swimming centres from the outskirts of Sheffield (*n* = 8), inability to perform swimming exercise (12), family commitments (*n* = 6), and reluctance to commit to the exercise intervention (*n* = 3), giving a recruitment rate of 43.5%.

### Feasibility of exercise

Compliance to the twice and thrice weekly sessions in an 8-week swimming exercise program was 92.6% and 88.4%, respectively, with no dropouts. In contrast, three participants were lost to follow-up assessment in the control group (no reasons were provided). No adverse events were reported during the swimming exercise.

### Side effects

None of the participants experienced side effects or adverse events due to their participation in the intervention.

### Quality of life

Quality of life as expressed by the EQ-5D-5L and VAS questionnaires demonstrated significant improvements in anxiety and VAS score for the swimming exercise compared to the control group following the 8-week exercise programme (Table 3). In addition, the control group presented a significant increase in anxiety at 8 weeks compared to baseline (Table 3).

## Discussion

The findings of the current study demonstrated that an 8-week swimming exercise intervention may improve body composition, as well as micro- and macrovascular function in older adults. More specifically, our exercise group experienced a decrease in WC, which constitutes a vital independent CV-risk factor (Ross et al., 2020). Our finding agrees with that of previous research, which explored the effects of a 6-week aquatic high-intensity interval training (HIIT) and moderate-intensity continuous training (MICT) programme (Tang et al., 2022a). Both exercise protocols demonstrated significant reductions in BMI, fat mass, and waist-to-hip ratio (Tang et al., 2022a). Moreover, in the same study, both exercise groups experienced an improvement in brachial artery FMD. This is in agreement with the current findings, which are based on a longer intervention. These findings, combined with our work focusing on microvascular function (Klonizakis et al., 2020), suggest that aquatic exercise may prevent age-related vasculopathy in older adults. The exploration of clinical benefits should be part of the next research step, defining the wider clinical application of swimming exercise.

The present study shows that 8 weeks of swimming exercise improved endothelial function in inactive adults. Principally, the exercise-induced adaptation of arterial stiffness is attributed to improved endothelial function and an increased rate of nitric oxide (NO) utilisation. During exercise, the mechanical effects on the arteries by virtue of repetitive exposure to the increases in blood pressure, blood flow, and arterial shear lead to increased NO production in endothelial cells, which promotes vascular smooth muscle relaxation (Green and Smith, 2018). Current evidence indicates that HIIT results in higher intensity of laminar shear stress compared to MICT, and thus, HIIT could induce a greater response in NO production (Adams et al., 2017). Nevertheless, our study did not directly record the swimming exercise intensity of our participants. Based on our previous experience and findings (Klonizakis et al., 2020), we can speculate that due to the inactive cohort’s low baseline fitness and swimming efficiency (i.e., lack of a skilled swimming technique), their swimming intensity was likely to be relatively high (e.g., HIIT). Our results indirectly suggest that an improvement in NO production depends on different exercise modalities. This may suggest that higher-intensity swimming exercise can induce effectively greater changes in vascular and, especially, endothelial function. Therefore, our results demonstrate that swimming exercise can improve both the endothelial function and structure, expressed by an increase in FMD response. Future studies shall explore the swimming intensities of inactive, amateur, older adult swimmers to comprehend the underlying physiological mechanisms of this vascular improvement. In addition, it would be useful to explore the haematological vascular markers as a response to the swimming exercise in older adults. The overall rating of participants’ health (i.e., VAS score) was significantly improved at the end of the swimming intervention compared to the control group. This finding agrees with previous evidence, which demonstrated that swimming exercise could improve lower- and upper-body strength and flexibility, functional mobility, and balance, while it can also reduce pain and increase QoL perceptions (Moreira et al., 2020). In addition, our study suggests that anxiety levels were significantly better after swimming than those in the control group, post-intervention. A recent systematic review supports our findings, indicating that swimming exercise could significantly improve mental health and anxiety symptoms (Tang et al., 2022b).

Evidently, the high rates of compliance, adherence, and retainment to the 8-week swimming exercise programme are an encouraging sign of the acceptability of our exercise intervention, aiming at the improvement of vascular function in older adults. Our findings indicate that swimming exercise is a well-preferred modality for older adults. Indeed, a recent systematic review reported that swimming exercise offers numerous psychological benefits in older adults, such as quality of life, mood, depression, anxiety, tension, and fall efficacy (Yoo, 2020). Nevertheless, the exact psychological motives that would allow us to structure better swimming interventions in older adults are still unknown. Future research needs to explore the enjoyment levels during the actual swimming exercise session, as well as the individual’s experiences and preferences from the post-swimming intervention (via interviews).

Swimming exercise has proven to be a feasible and effective exercise programme in older adults. Our next step will be the implementation of a large-scale clinical trial. This trial could explore the health economics (i.e., NHS burden alleviation) of a long-term intervention (i.e., >12 weeks), alongside physiological outcomes, and individual preferences in order to establish its utility as a pragmatic community service. The results will aim to inform and create a commissioned service that community GPs and/or older adults could be referred or self-referred to, respectively.

## Limitations

Although we obtained some promising results in the present study, there were some limitations, particularly around the sample size of the study. Nevertheless, we need to consider that our study aimed to provide preliminary evidence for a larger clinical trial and that it was also conducted amid strict social restrictions (i.e., swimming pool closure periods) due to the COVID-19 pandemic. Another limitation to our study is that we did not record the aqua exercise intensity (e.g., via water-resistant heart rate monitors), and thus, we were not able to explain some of the physiological mechanistics. Nevertheless, we conducted a pragmatic intervention rather than a physiology-based exercise protocol. In a pragmatic swimming environment, an amateur, older swimmer will not have the experience to alter their pace and, thus, their intensity, nor to swim efficiently—this is something that only experienced swimmers can perform. In addition, and based on our previous findings from the ACELA I study, those who perform swimming exercise regularly (i.e., 3–4 times per week) for a long term (i.e., ≥6 months), regardless of the exercise intensity that they followed, demonstrated an improved vascular function compared to sedentary counterparts (Klonizakis et al., 2020). Therefore, further studies on a larger scale and for a longer training period (i.e., >3 months) should be conducted, focusing on clinical outcomes and assessing the clinical and cost effectiveness of a swimming intervention.

## Conclusion

Swimming exercise is an effective modality to improve the vascular function and overall QoL in older adults. Moreover, our 8-week, community-based, self-managed, pragmatic swimming exercise intervention is feasible and could be implemented in older adults for the prevention of age-related cardiovascular disease. We reported improved endothelial function at both micro- and macrocirculatory levels for the swimming exercise group compared to the sedentary group. These findings suggest that swimming exercise could significantly reduce CVD risk in older adults. A large research clinical trial is warranted to establish these findings and assess the clinical and cost effectiveness of the intervention.

## Data Availability

The datasets presented in this article are not readily available due to ethical constraints. Requests to access the datasets should be directed to MK.
